# The effect of postoperative anticoagulation on false lumen patency after surgery for acute type A aortic dissection

**DOI:** 10.1186/s13019-021-01661-1

**Published:** 2021-09-28

**Authors:** Mårten Larsson, Gracijela Bozovic, Johan Sjögren, Igor Zindovic, Sigurdur Ragnarsson, Shahab Nozohoor

**Affiliations:** 1 Department of Clinical Sciences Lund, Cardiothoracic Surgery, Lund University and Skåne University Hospital, Getingevägen 4, 221 85 Lund, Sweden; 2 Department of Medical Imaging and Clinical Physiology, Lund University and Skåne University Hospital, Lund, Sweden

**Keywords:** Aortic dissection, False lumen, Anticoagulation

## Abstract

**Background:**

Patent false lumen has been shown to have a negative impact on prognosis after surgery for acute type A aortic dissection (ATAAD). We aimed to assess the effect of postoperative anticoagulation on false lumen patency and clinical outcomes in relation to false lumen status.

**Methods:**

Postoperative computed tomographies of 156 patients undergoing ATAAD DeBakey type I surgery were retrospectively evaluated for false lumen patency. The patients were divided into groups determined by anticoagulation treatment at discharge. Uni- and multivariable logistic regression was used for analysing the effect of anticoagulation on the false lumen, and Kaplan–Meier estimates were used to assess the association of a patent false lumen with the incidence of reoperation and long-term survival.

**Results:**

A patent false lumen was present in 81% of the patients. Postoperative anticoagulants were not associated with a patent false lumen (*p* = 0.48) in univariable analysis. In multivariable analysis, both hemiarch replacement (OR 0.15, CI95% 0.05–0.49, *p* = 0.001) and the use of betablockers had a protective effect (OR 0.29, CI95% 0.10–0.85, *p* = 0.023). The Kaplan–Meier estimates for survival and the composite endpoint of survival and freedom from distal reintervention indicated no difference in outcome between patients in regard to anticoagulation treatment (survival *p* = 0.43, composite *p* = 0.82*)* or false lumen status (survival *p* = 0.21, composite *p* = 0.09).

**Conclusion:**

This study could not show negative effects from the postoperative use of anticoagulants on false lumen status, nor that false lumen patency was associated with poorer prognosis. A hemiarch procedure was shown to be associated with reduced risk of false lumen patency.

## Introduction

Acute type A aortic dissection (ATAAD) requires emergent surgical repair primarily to prevent ascending aortic rupture, cardiac tamponade, acute aortic valve insufficiency with consequent ventricular dysfunction, and cerebral malperfusion with associated stroke or coma. Ideally, surgical treatment reestablishes blood flow through the true lumen thereby depressurizing and decreasing the false lumen. Shrinkage and thrombosis of the false lumen, as well as remodeling of the aorta also are desirable but not always achieved. In spite of successful repair of the proximal entry tear, the true lumen may fail to adequately dilate distally. A patent false lumen has been shown to be associated with an increased risk of late mortality, enlargement of the remaining aorta, and reintervention aimed at the distal aorta(1–4). In analogy, studies of type B dissection have suggested that patients with complete thrombosis of the false lumen have improved outcomes, whereas those with a patent false lumen have an increased risk of aortic enlargement and death [1–3].

Anticoagulation therapy after surgery for ATAAD is indicated if the patient has a mechanical valve implantation or atrial fibrillation. However, the use of anticoagulants could theoretically lead to lower rates of false lumen thrombus formation, one of the secondary goals of surgery. If there is a substantial risk of reoperation or death, the small risk of thromboembolism in case of atrial fibrillation might be justified. And in the setting of determining which valve to implant, the benefit of a biological valve without demand for anticoagulation might justify implantation of this valve at a lower age than normally recommended. The rate of false lumen thrombus formation in relation to anticoagulation therapy has been studied previously, and these studies indicate similar or higher rates of false lumen patency in patients using anticoagulants [4–6].

The present study aimed to evaluate if anticoagulation postoperative have an effect on false lumen patency and to study the effect of postoperative false lumen patency on survival and the rate of reintervention in patients presenting with ATAAD DeBakey type I.

## Methods

This was a single centre, retrospective, observational study and included 347 patients who underwent surgery for ATAAD from January 2005 to December 2018 at the Department of Cardiothoracic Surgery, Skane University Hospital, Lund, Sweden. Pre-, peri-, and postoperative variables were prospectively collected and entered into the department’s computerised database for retrospective analysis, and medical records were reviewed when necessary. Survival data was obtained from the Swedish National Board of Health and Welfare, (*Socialstyrelsen, Sweden*). The registry was approved by the regional ethical review board, and a waiver of informed consent for the retrospective review of medical records was granted for the registry (ref. 2015/197).

**Fig. 1:**
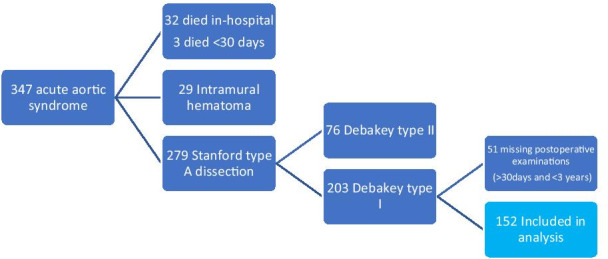
Flow chart of patients with acute aortic syndromes referred to the Department of Cardiothoracic Surgery, Skåne University Hospital, Lund, during the inclusion period

Patients who died during hospital admission or within 30 days after surgery were excluded from the analysis. The preoperative computed tomography (CT) scans of the aorta (mostly non-ECG gated) with and without intravenous contrast enhancement of 347 patients were examined on a clinical Picture Archiving and Communication System (PACS) workstation. Intramural hematoma (IH) defined as a focal, crescent-shaped, high attenuating (60–70 Hounsfield unit, HU) region in an eccentrically thickened aortic wall on a non-contrast-enhanced CT was present in 29 patients, and in four patients the distinction between IH and ATAAD was not possible to establish [7], leading to exclusion. An additional 76 patients presented with DeBakey type II ATAAD and were excluded as the false lumen can be totally resected in most cases, leaving little to no risk of false lumen patency. Of the remaining 232 patients, the postoperative CT scans performed within one year were evaluated. Another 51 patients did not have any postoperative CTs registered and were thus excluded. Finally, a cohort of 156 patients remained for assessment. The flow chart of the inclusion protocol is presented in Fig. 1. All examinations were evaluated at two separate occasions to reduce the risk of bias.

### Endpoints

The primary endpoint was the association between the postoperative anticoagulation regimen and the incidence of patent false lumen one year after surgery or the last known imaging prior to or after one year. Secondary endpoints were death or the composite of death and reintervention (open or endovascular surgery to the aorta or its immediate branches > 24 h after initial surgery) due to a patent false lumen or anticoagulation. Anticoagulation treatment was established at discharge from hospital. Patients treated with warfarin or non-vitamin K antagonist oral anticoagulants (NOACs) were defined as the anticoagulation group and compared to the remaining patients constituting the control group. Major bleeding was defined based on the BART criteria with patients fulfilling any of these variables: postoperative bleeding through chest tubes exceeding 1500 ml over any 8-h period; reoperation for bleeding or cardiac tamponade within 24 h of surgery; transfusion of more than 10 U of red blood cells within 24 h after surgery; or death from haemorrhage within 30 days.

### Radiology

The CT examinations of the aorta were performed with intravenous contrast in routine scan settings and reconstruction parameters at our institution and referring hospitals with a diverse machine park ranging from Philips Mx8000 Idt 16 to Dual Source CT Scanner, reflecting the rapid development of CT scanners during the study period. The images varied from 5 mm thick slices reconstructed in three planes to 1 mm source slices enabling complete reconstructions. All postoperative contrast-enhanced CT scans of the aorta within one year were evaluated for persisting false lumen [8] on a clinical PACS workstation with an appropriate window setting for the aorta [9]. False lumen was considered patent if any contrast was found outside the true lumen in the thoracic aorta. The patients were divided into three groups according to the status of their aorta. Group I patients showed no contrast outside the true lumen, group II had a patent false lumen in either ascending and/or arch (including or excluding descending aorta) and group III had a patent false lumen only in the descending aorta. All postoperative CT scans involving the chest with contrast enhancement within one year were evaluated. For patients with a limited number of examinations, the examination closest to one year with > 30 days follow-up was chosen.

### Surgical procedure

As previously described [10], surgery was performed through a median sternotomy with cardiopulmonary bypass. Deep hypothermia (< 20ׄ°C) and circulatory arrest was used in most cases with open distal anastomosis after inspection of the ascending aorta and the aortic arch. Cerebral protection with steroids (500 mg methylprednisolone) and a barbiturate (thiopental or phenobarbital) was used routinely in deep hypothermic arrest. In eight cases, cross-clamp was used in normothermia. After distal anastomosis was completed, distal perfusion was re-established through a side branch in the vascular prosthesis, the patient rewarmed, and the proximal aorta, valve and root inspected for tears. If deemed necessary, the valve and/or or root was replaced. When the proximal anastomosis was completed and the patient was normothermic, cardiopulmonary bypass was terminated.

### Follow-up

Surgical or endovascular treatment during follow-up was considered if any of the following were observed: dissection progression with signs of impending rupture, signs of impaired visceral or peripheral perfusion, retrograde extension of the dissection to the remaining arch or ascending aorta, increasing aortic insufficiency, and aortic diameter > 55 mm.

### Statistics

Categorical data were given as proportions, and continuous variables were expressed as the mean ± standard deviation. In skewed distributions, medians and interquartile ranges (IQRs) were reported. Proportions were compared using the chi-square test. If the expected frequency was < 5, Fisher’s exact test was applied. For continuous variables, Student’s *t*-test was used. Event rates during follow-up were estimated and survival was plotted using the Kaplan–Meier method, with the differences between groups compared using the log-rank test. Uni- and multivariable logistic regression analyses were performed to determine independent predictors of patent false lumen and relied on complete cases analysis. Variables that were considered to potentially impact false lumen status and with a *p* value < 0.1 were fitted to a multivariable regression model. Postoperative treatment with anticoagulation was forced into the analysis. The results of logistic regression analyses are expressed as hazard ratios (HRs) and odds ratios (ORs) with 95% confidence intervals (CI). A *p* value < 0.05 was considered statistically significant unless otherwise stated. Statistical analysis relied on standard software (IBM Corp. Released 2017. IBM SPSS Statistics for PC, Version 25.0.0. Armonk, NY, USA: IBM Corp.)

## Results

The mean follow-up for mortality was 6.5 ± 3.9 years (median 5.6 years; IQR 3.2‒8.3). Mean follow-up for the composite endpoint of mortality and reintervention was 5.2 ± 3.5 years (median 4.5 years; IQR 2.5‒7.5) and mean follow-up of the last CT was 358 ± 184 days, (median 389 days; IQR 268–475).

**Fig. 2:**
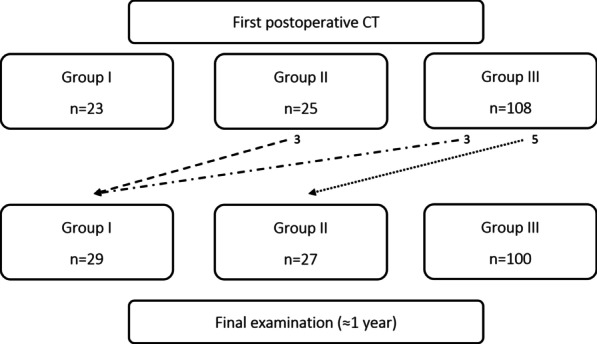
Flow chart of false lumen status and change during the observation period

A patent false lumen was present in 81% of patients (n = 127/156) with 17% (n = 27) being categorised as group II and 64% (n = 100) as group III at 1-year follow-up. In 7% of patients (n = 10), the false lumen group changed during the observation period (Fig. 2). At discharge, 36% of patients (n = 56) received anticoagulation treatment, 18 patients were treated with NOAC, and 38 patients received warfarin of which one was treated 3 months postoperatively due to biological valve prosthesis (institution protocol at the time) and all others were indefinite due to atrial fibrillation or mechanical valve prosthesis. The control group (n = 100) consisted of patients who did not receive anticoagulation treatment (n = 45), received low dose aspirin (75 mg) (n = 51), or received other treatments (n = 4). Of these, one patient received aspirin and dipyridamole, one low dose low molecular weight heparin (LMWH), one aspirin and low dose LMWH, and one full dose LMWH. None of the patients on LMWH were treated longer than 3 months postoperatively. During the inclusion period, the in-hospital mortality was 13.6% (n = 39) and surgical mortality (30-day or in-hospital mortality) was 13.9% (n = 40) for all patients undergoing surgery for ATAAD (n = 287).

The perioperative patient characteristics in relation to anticoagulation therapy are presented in Tables 1 and 2. Patients with anticoagulation treatment were younger (59.0 ± 13.3 vs 61.9 ± 9.3, *p* = 0.01), less frequently treated with ACE-inhibitors preoperatively (14% vs 30%, *p* = 0.03), and were more often operated with aortic root replacement procedure (62% vs 10%, *p* < 0.001).

**Table 1:** Preoperative characteristics

	No anticoagulation (n = 100)	Warfarin or NOAC (n = 56)	p-value
Patent false lumen	79 (79%)	48 (86%)	0.39
Age (years)	61.9 ± 9.3	59.0 ± 13.3	0.01
Female	27 (27%)	17 (30%)	0.71
Hypertension	52 (52%)	23 (41%)	0.19
Thoracic aneurysm	9 (9%)	5 (9%)	1
Marfan	7 (7%)	5 (9%)	0.67
Other connective disease	1 (1%)	1 (2%)	1
BAV	2 (2%)	2 (4%)	0.62
PVD	1 (1%)	1 (2%)	1
DM	25 (25%)	8 (14%)	0.12
Hyperlipidemia	5 (5%)	2 (4%)	1
Stroke	4 (4%)	2 (4%)	1
CKD	1 (1%)	1 (2%)	1
COPD	7 (7%)	1 (2%)	0.26
CAD	6 (6%)	1 (2%)	0.42
Smoking history	30 (30%)	14 (25%)	0.48
BMI	26.1 ± 3.9	27.1 ± 4.5	0.18
Betablocker	18 (18%)	8 (14%)	0.52
ACE inhibitor	29 (30%)	8 (14%)	0.03
Systolic blood pressure (mmHg)	127 ± 35	118 ± 32	0.18
Diastolic blood pressure (mmHg)	72 ± 21	65 ± 17	0.08

Values are expressed as numbers (%) or mean ± SD

*BAV* bicuspid aortic valve, *PVD* periferal vascular disease, *DM* diabetes mellitus, *CKD* chronic kidney disease, *COPD* chronic obstructive pulmonary disease, *CAD* coronary artery disease, *BMI* body mass index, *ACE* angiotensin converting enzyme

**Table 2:** Intra- and postoperative characteristics

	No anticoagulation (n = 104)	Warfarin or NOAC (n = 52)	*p* value
Proximal surgery			< 0.01
Supracoronary graft	85 (85%)	19 (34%)	
Bentall procedure	9 (9%)	33 (59%)	
Mechanical	0	28 (54%)	
Bioprosthesis	9 (9%)	5 (9%)	
David/Yacoub	4 (4%)	0	
AVR and supracoronary graft	2 (2%)	4 (7%)	
Distal surgery			0.55
Ascending aortic graft	77 (77%)	46 (82%)	
Hemiarch	15 (15%)	5 (9%)	
Arch	8 (8%)	5 (9%)	
Crossclamp	5 (5%)	3 (5%)	1
Circulatory arrest	50 (48%)	29(56%)	0.07
Retrograde cerebral perfusion	42 (40%)	19 (37%)	
Antegrade cerebral perfusion	11(11%)	1(2%)	
Primary tear excised	62 (62%)	36 (66%)	0.67
GRF Glue	65 (65%)	29 (52%)	0.11
CPB time (min)	192 ± 54	218 ± 60	0.28
HCA time (min)	24 ± 10	23 ± 10	0.39
Arterial cannulation			0.11
Femoral	75 (75%)	50 (89%)	
Subclavia	3 (3%)	2 (4%)	
Ascending aorta or arch	19 (19%)	4 (7%)	
Left ventricle	3 (3%)	0	
Reop bleeding	11 (11%)	1 (2%)	0.06
Plasma (units)	4.5 ± 4.4	3.7 ± 4.3	0.32
Platelets (units)	3.6 ± 2.8	3.5 ± 2.2	0.97
Red blood cells (units)	5.5 ± 5.1	4.1 ± 3.9	0.07
Fibrinogen^a^ (g)	5.2 ± 3.4	5.0 ± 2.6	0.79
Recombinant FVII (mg)	1.8 ± 2.4	1.8 ± 3.0	0.98
Recombinant FVII	23 (39%)	11 (30%)	0.39
Bleeding 24 h (ml)	860 ± 660	702 ± 306	0.17
Massive bleeding^b^	23 (23%)	6 (11%)	0.06

Values are expressed as numbers (%) or mean ± SD

*AVR* aortic valve replacement, *GRF* Gelatin Resorcinol Formaldehyde, *CPB* cardiopulmonary bypass, *HCA* hypothermic circulatory arrest

^a^60 missing values (38%)

^b^According to BART criteria

Patient and operative characteristics in relation to closed (group I) or patent false lumen (group II + III) are presented in Tables 3 and 4. The use of a hemiarch procedure (*p* = 0.03) and preoperative treatment with betablocker (*p* = 0.02) reduced the risk of patent false lumen in univariable analysis. The need for transfusion with platelets as well as fibrinogen supplement was higher in the patent false lumen group (*p* = 0.03 and *p* = 0.01, respectively). However, transfusion with red blood cells or plasma did not differ between groups nor did rates of reoperation for bleeding or massive bleeding.

**Table 3:** Preoperative characteristics

	Closed false lumen (n = 29)	Patent false lumen (n = 127)	*p* value
Age (years)	63.6 ± 11.6	60.2 ± 10.7	0.14
Female	12 (41%)	32 (25%)	0.08
Hypertension	15 (52%)	60 (47%)	0.70
Thoracic aneurysm	3 (10%)	11 (9%)	0.73
Marfan	3 (10%)	9 (7%)	0.55
Other connective disease	0	2 (2%)	
BAV	0	4 (3%)	
PVD	1 (3%)	1 (1%)	0.34
DM	8 (28%)	25 (20%)	0.35
Hyperlipidemia	2 (7%)	5 (4%)	0.62
Stroke	0	6 (5%)	0.59
CKD	0	2 (2%)	
COPD	4 (14%)	3 (3%)	0.04
CAD	3 (10%)	4 (3%)	0.12
Smoking history	7 (24%)	37 (29%)	0.57
BMI	25.8 ± 4.8	27.1 ± 4.9	0.20
Betablocker	9 (28%)	17 (14%)	0.02
ACE inhibitor	8 (28%)	29 (23%)	0.62
Systolic blood pressure (mmHg)	125 ± 35	124 ± 34	0.83
Diastolic blood pressure (mmHg)	73 ± 20	69 ± 21	0.38

Values are expressed as numbers (%) or mean ± SD

*BAV* bicuspid aortic valve, *PVD* periferal vascular disease, *DM* diabetes mellitus, *CKD* chronic kidney disease, *COPD* chronic obstructive pulmonary disease, *CAD* coronary artery disease, *BMI* body mass index, *ACE* angiotensin converting enzyme

**Table 4:** Intra- and postoperative characteristics

	Closed false lumen (n = 29)	Patent false lumen (n = 127)	*p* value
Proximal surgery			0.09
Supracoronary graft	23 (79%)	81 (64%)	
Bentall procedure	4 (14%)	38 (30%)	
Mechanical	3 (10%)	25 (20%)	
Bioprosthesis	1 (3%)	13 (10%)	
David/Yacoub	1 (3%)	4 (3%)	
AVR and supracoronary graft	1 (3%)	3 (4%)	
Distal surgery			0.03
Ascending aortic graft	19 (66%)	104 (82%)	
Hemiarch	8 (27%)	12 (9%)	
Arch	2 (7%)	11 (9%)	
Crossclamp	4 (9%)	4 (4%)	0.21
Primary tear excised	20 (69%)	78 (62%)	0.48
GRF Glue	19 (66%)	75 (59%)	0.52
CPB time (min)	191 ± 43	204 ± 60	0.28
HCA time (min)	21.5 ± 8.8	23.5 ± 11.0	0.28
Arterial cannulation			0.86
Femoral	24 (83%)	101 (80%)	
Subclavia	1 (3%)	4 (3%)	
Ascending aorta or arch	4 (14%)	19 (15%)	
Left ventricle	0	3 (2%)	
Reop bleeding	1 (3%)	11 (9%)	0.47
Plasma (units)	3.9 ± 5.4	4.3 ± 4.1	0.72
Platelets (units)	2.6 ± 2.1	3.5 ± 2.7	0.03
Red blood cells (units)	5.6 ± 6.5	4.8 ± 4.3	0.44
Fibrinogen^a^ (g)	3.2 ± 2.0	5.4 ± 3.2	0.01
Recombinant FVII (mg)	1.3 ± 2.3	1.9 ± 2.7	0.46
Recombinant FVII	3 (20%)	31 (38%)	0.24
Bleeding 24 h (ml)	673 ± 370	853 ± 524	0.17
Massive bleeding^b^	5 (17%)	24 (19%)	0.84
Anticoagulation			
NOAC	4 (14%)	14 (11%)	0.75
Warfarin	8 (17%)	31 (29%)	0.12

Values are expressed as numbers (%) or mean ± SD

*AVR* aortic valve replacement, *GRF* gelatin resorcinol formaldehyde, *CPB* cardiopulmonary bypass, *HCA* hypothermic circulatory arrest

^a^60 missing values (38%)

^b^According to BART criteria

The multivariable model presented in Table 5 showed that betablocker treatment (OR 0.24, CI 95% 0.08–0.68, *p* = 0.007) and hemiarch replacement (OR 0.18, CI 95% 0.06–0.56, *p* = 0.003) were associated with a reduced risk of false lumen patency whereas platelet transfusion was an independent predictor of false lumen patency (OR 1.30, CI 95% 1.03–1.64, *p* = 0.03).

**Table 5:** Multivariable logistic regression analysis evaluating predictors of a patent false lumen

	Multivariate logistic regression		
	OR	CI95%	*p* value
Betablocker	0.24	0.08–0.68	0.007
Distal surgery:			
Supracoronary graft	1		
Hemiarch^a^	0.18	0.06–0.56	0.003
Arch^a^	0.83	0.16–4.3	0.82
Platelets (units)	1.30	1.03–1.64	0.03
Age			ns
Sex			ns
CAD			ns
Anticoagulation			ns

*CAD* coronary artery disease

^a^Compared to supracoronary graft

During follow-up, 25 patients had surgical or endovascular reinterventions. Eighteen patients underwent open thoracic surgery, 12 on the distal aorta (6 on the aortic arch, 7 on the descending aorta) and 11 on the proximal aorta. Eight patients had endovascular surgery, three of whom were treated with thoracic endovascular aortic repair (TEVAR) and two with fenestrated endovascular aortic repair (FEVAR). The remaining three patients were treated with stents in the mesenteric artery, the renal arteries, or the internal iliac artery. There were two deaths associated with these reoperations, one of whom presented with rapid dilatation of the proximal descending aorta and suspected infection or aortitis and had open arch surgery, and one of whom had been operated initially for ATAAD with a biological valve and root replacement and was later reoperated with a homograft due to endocarditis and a proximal descending aortic aneurysm of eight cm requiring replacement with a frozen elephant trunk.

**Fig. 3:**
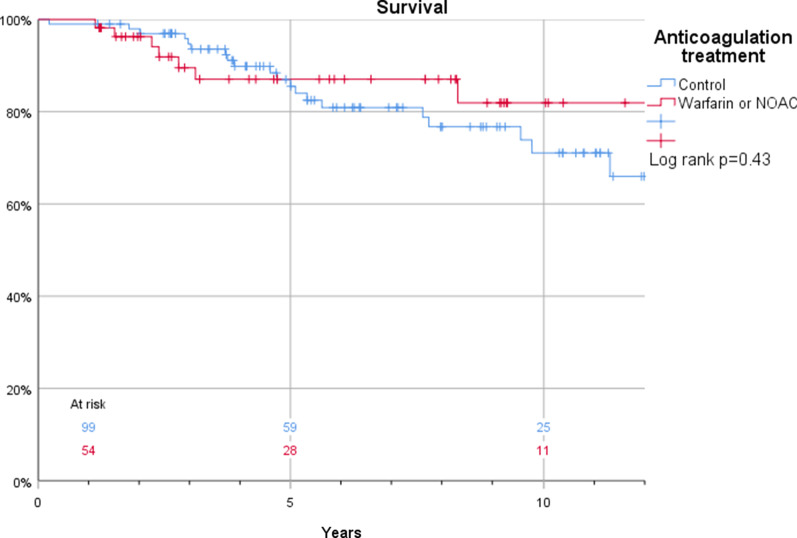
Kaplan–Meier estimates of survival plotted for anticoagulation treatment

**Fig. 4:**
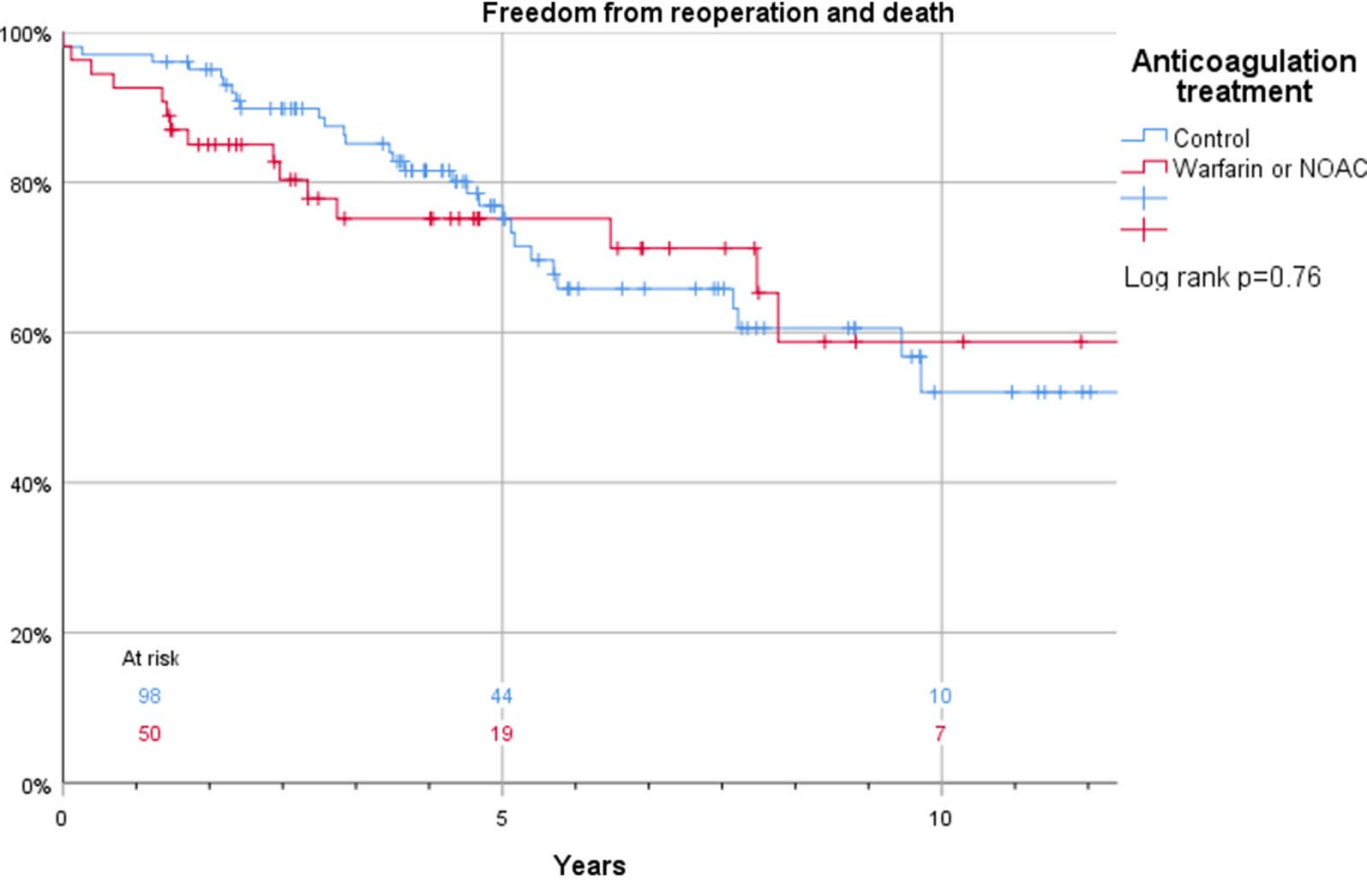
Kaplan–Meier estimates of the composite endpoint of survival and freedom of reintervention plotted for anticoagulation treatment

**Fig. 5:**
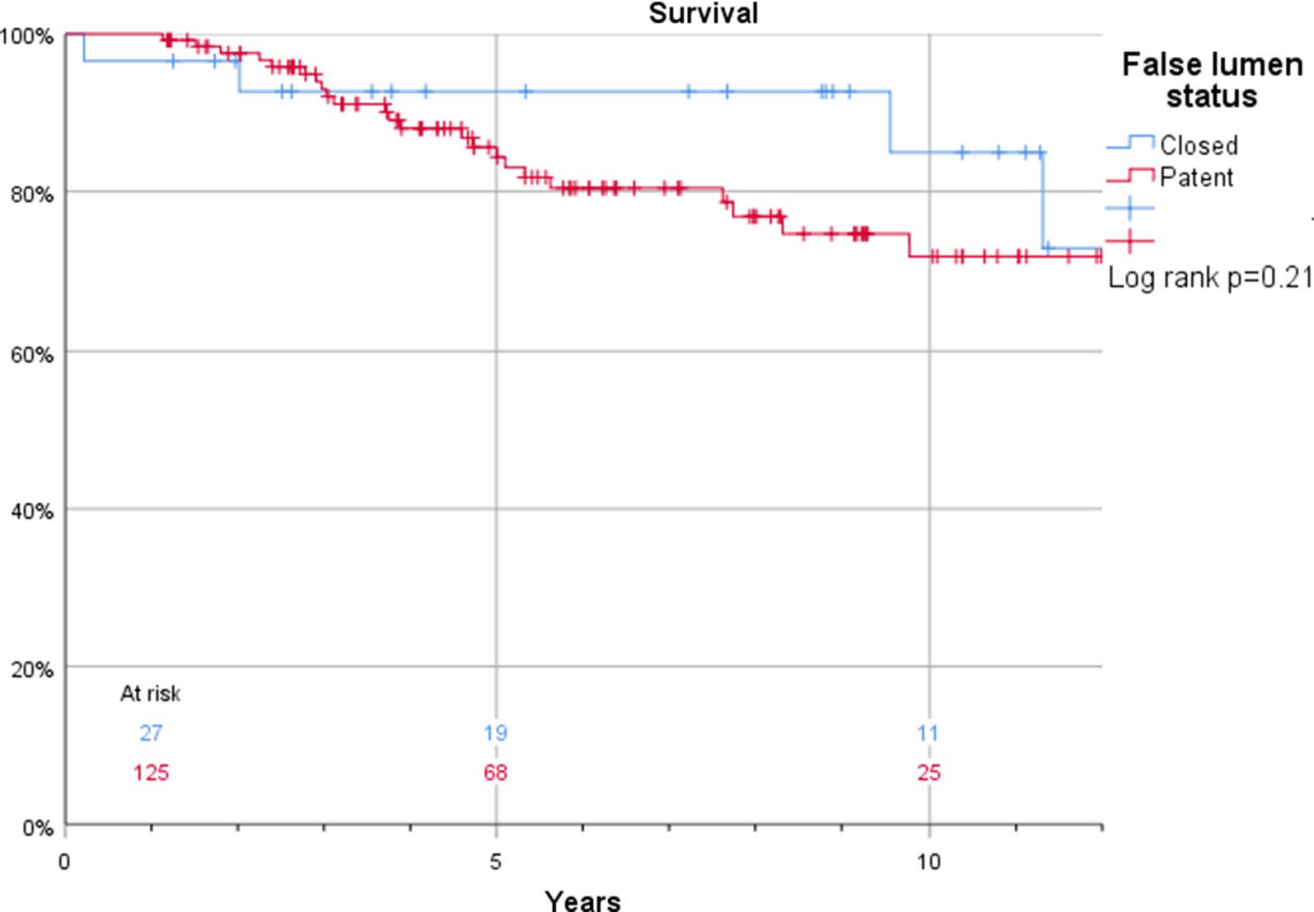
Kaplan–Meier estimates of survival plotted for false lumen status

**Fig. 6:**
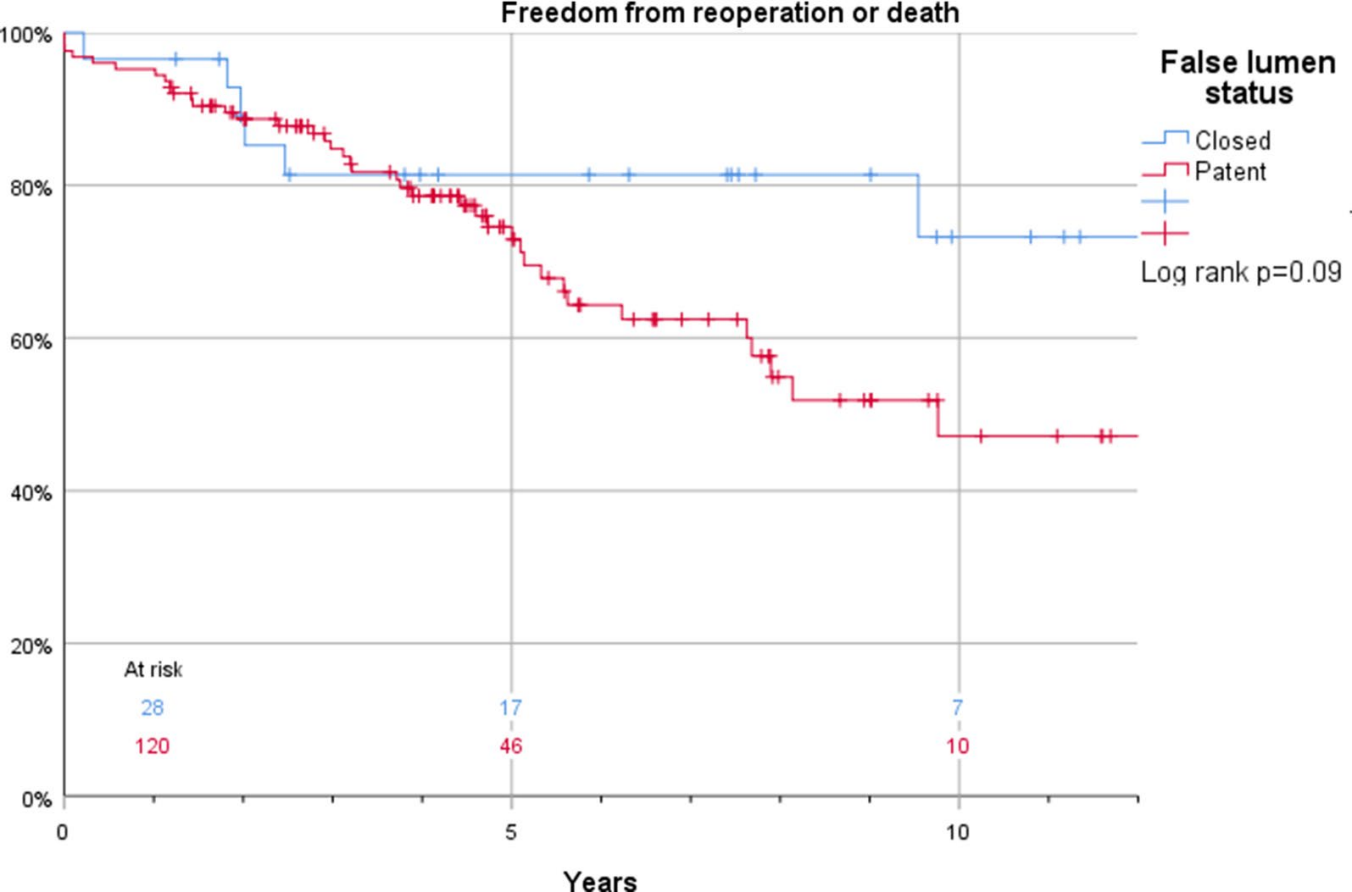
Kaplan–Meier estimates of the composite endpoint of survival and freedom of reintervention plotted for false lumen status

**Fig. 7:**
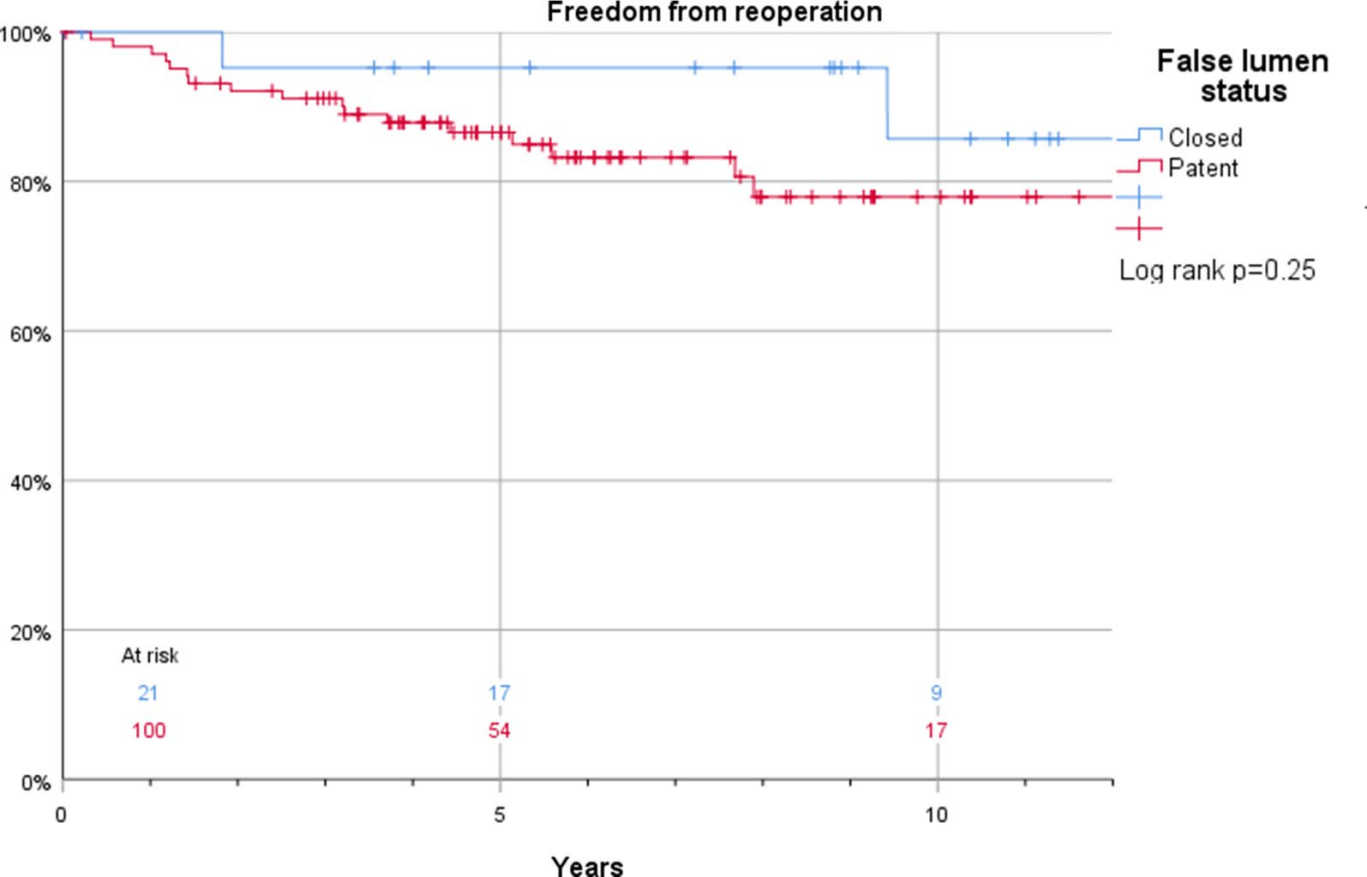
Kaplan–Meier estimates of freedom from reoperation plotted for false lumen status

The Kaplan–Meier estimates for survival in patients who were discharged and thus included in this study were 100% vs. 99%, 96% vs. 98%, and 88% vs. 85% at 1, 2, and 5 years, respectively (log rank *p* = 0.43) in patients with or without anticoagulant treatment (Fig. 3). Corresponding estimates for the composite endpoint of survival and freedom of reintervention were 94% vs. 96%, 86% vs. 90%, and 76% vs. 75% at 1, 2, and 5 years, respectively (log rank *p* = 0.76) (Fig. 4). Also, false lumen status did not have a significant impact on survival (97% vs. 100%, 97% vs. 98%, and 93% vs. 84%) at 1, 2, and 5 years, respectively in patients with a patent and occluded false lumen (log rank *p* = 0.21) (Fig. 5). Neither did the composite endpoint of survival and freedom of reintervention (96 vs 94%, 90 vs 86% and 75 vs 76% at 1, 2, and 5 years, respectively, log rank *p* = 0.09) (Fig. 6). Freedom from reoperation did not differ between groups (100 vs 98%, 95 vs 92% and 95 vs 87% at 1, 2, and 5 years, respectively, log rank *p* = 0.25) (Fig. 7).

## Discussion

In the present study, anticoagulation treatment had no significant impact on the incidence of a false lumen patency within the first postoperative year after surgery for ATAAD. Postoperative beta-blocker treatment reduced the risk of patent false lumen, but false lumen patency was neither associated with impaired survival nor the need for reintervention.

In the current study, we hypothesized that postoperative anticoagulation therapy maintain a patent false lumen.Data in the present study showed that anticoagulation treatment was not associated with a higher incidence of patent false lumen, but in contrast to a report by Song et al. [4] who investigated 118 patients with DeBakey type I ATAAD, we could not demonstrate improved survival for patients treated with anticoagulants. In this study, patients receiving anticoagulation were younger and primarily treated with warfarin due to implantation of a mechanical valve in a root replacement procedure, which intuitively should result in longer life expectancy. Although there are no current recommendations regarding the use of anticoagulation following ATAAD repair, many physicians believe anticoagulation may have a negative effect on remodeling of the residual distal aorta. However, our data supports the findings of Song et al. [4] that anticoagulation is not associated with a reduced rate of false lumen thrombosis. This is further supported by the findings by von Kodolitsch et al. [8] presented in the WATAS study including 243 patients after ATAAD of which 106 patients were investigated with serial imaging. They found no difference in partial or fully patent false lumen with regards to warfarin treatment. However, Gariboldi et al. [9] investigated 147 patients with aortic dissection DeBakey type I-III of which 106 were DeBakey type I and could show a reduced probability of false lumen thrombosis if the patient received long-term oral anticoagulation (OR 0.31 (CI 95% = 0.10–0.84)).

The incidence of a patent false lumen during postoperative follow-up in the current study (81%) is similar to those presented previously with figures ranging from 47 to 74% [4, 6, 11–13]. It is possible that the low percentage of hemiarch and arch replacements in the present series (arch replacement 8%, hemiarch 13%) led to the relatively high rate of false lumen patency.

Suboptimal connection of the distal part of the graft implanted in the ascending aorta to the true lumen or the presence of secondary tears in the arch or distal aorta may account for the persistence of flow into the residual false lumen after complete surgical resection of the primary entry tear [14]. To decrease the incidence of residual patent false lumen, some authors suggest systematic extended or total arch replacement for the initial surgical management of ATAAD, irrespective of the site of entry [15, 16]. Those supporting this method claim improved long term survival [17], while others maintain no improvement in long term survival but rather increased early mortality [18]. In this study, 40% of the patients operated with hemiarch replacement had a patent false lumen compared to 84% for patients receiving a supracoronary graft. Hemiarch replacement seems to be protective against a patent false lumen at one year (OR 0.18, CI95% 0.06–0.56, *p* = 0.003), which supports the increasing trend towards this surgical technique [15, 16, 19]. Interestingly, arch replacement did not show similar results. However, the number of patients operated with this technique in this study was small and the in-hospital mortality higher (21% vs 15% for arch vs ascending/hemiarch replacement for all 283 patients with ATAAD), which is consistent with previous reports [20, 21].

Hypertension is a known risk factor for developing aortic aneurysm and ATAAD, and the use of betablockers has been shown to reduce the growth rate of aortic aneurysm. This study shows that treatment with betablockers also is beneficial after surgery for ATAAD, reducing the risk of a patent false lumen (OR 0.24, CI95% 0.08–0.68, p = 0.007), probably indicating the importance of strict postoperative blood pressure control.

The main hypothesis for this investigation was that anticoagulants increase postoperative false lumen patency. The reverse hypothesis could be that procoagulants reduce false lumen patency. Fibrinogen and recombinant factor VII have proved to reduce the need for transfusion in cardiac surgery [22, 23] but neither the use (*p* = 0.19, OR 2.48, CI95% 0.65–9.49) nor the administrated dose (*p* = 0.46, OR 1.09, CI95% 0.87–1.38) of recombinant factor VII had a significant impact on the false lumen status.

However, we did find a significant association between fibrinogen and a higher incidence of patent false lumen (*p* = 0.02, HR 1.38, CI95% 1.07–1.79) in univariable analysis, but due to numerous missing values, fibrinogen was not included in the multivariable model. Moreover, transfusion of platelets was also increased in patients with patent false lumen (OR 1.30, CI95% 1.03–1.64, *p* = 0.03). These contradicting findings may merely reflect a more severe coagulopathy, requiring larger doses of procoagulant products, with the severe coagulopathy maintained by the false lumen.

Late survival in 30-day survivors was 99%, 87%, and 74% at 1, 5, and 10 years, respectively, in this study, which is comparable to contemporary data from the NORCAAD registry [24]. Time from the index operation to reoperation has been reported to be approximately five years [6, 12]. In the present study, both mortality and the need for surgical/endovascular treatment increased from the third year of follow-up after ATAAD in all patients with persistent patent false lumen, which suggests that structural and/or dynamic factors responsible for dissection complications require time to develop.

The present study had several limitations. The series was not that large thus increasing the risk of type II errors and biases. However, this study population was homogenous and excluded DeBakey type II ATAAD and intramural hematomas. This study was performed retrospectively, and the indications for using anticoagulation differed. Therefore, the sole effect of the drugs on the degree of thrombosis or false lumen patency after surgical repair of ATAAD may not be generalizable. The main reason for missing examinations were due to patients referred from other regions whose examinations postoperatively are not routinely made available to the investigators. Patients in need of reoperation were most likely referred to our center due to regional agreements, lowering the risk of underestimating the need for reoperation. Additionally, the examiner was not blinded to anticoagulation, however, a mechanical valve is not possible to blind, and the fate of the false lumen was rarely hard to determine.

During the study period of thirteen years, substantial changes in routinely imaging surveys and advances in imaging techniques improved morphological and functional assessment. Some CT examinations were performed only in the early arterial phase, which might have led to an underestimation of the actual incidence of residual patent false lumen. Moreover, enhanced CT scanning was not routinely performed in all patients who were included, and no assessment of growth rate or partial thrombosis was performed.

Although our surgical approach may have led to a relatively high incidence of residual patent false lumen, the long-term outcomes were acceptable and did not differ according to the status of the residual false lumen.

## Conclusion

Anticoagulation treatment had no impact on the incidence of a false lumen over the first postoperative year. Mechanical valve is a feasible option for younger patients and anticoagulation for atrial fibrillation may be used with no regard to false lumen patency in surgery for ATAAD. Hemiarch replacement seems to be favorable in reducing the rate of a patent false lumen and support the current opinion that this should be the gold standard for ATAAD repair [25, 26]. Nevertheless, a careful follow-up after surgery for ATAAD is still necessary for all patients.

## Data Availability

Data not available for review due to limitations in ethics approval.

## Abbreviations

ATAAD: Acute type A aortic dissection
CT: Computed tomography
PACS: Picture archiving and communication system
IH: Intramural hematoma
HU: Hounsfield unit
NOAC: Non vitamin-K antagonist oral anticoagulant
IQR: Interquartile range
HR: Hazard ratio
OR: Odds ratio
CI: Confidence interval
LMWH: Low molecular weight heparin
ACE: Angiotensin converting enzyme
TEVAR: Thoracic endovascular aortic repair
FEVAR: Fenestrated endovascular aortic repair
